# CC-Type Glutaredoxin *MeCEPD* Functions as an Important Regulatory Component in Response to Nitrate Starvation in Cassava

**DOI:** 10.3390/plants15071056

**Published:** 2026-03-30

**Authors:** Xiaochen Liu, Bo Liu, Yunpeng Dai, Weitao Mai, Wenquan Wang, Changying Zeng, Xin Chen

**Affiliations:** 1 School of Tropical Agriculture and Forestry/Sanya Institute of Breeding and Multiplication, Hainan University/State Key Laboratory of Tropical Crop Breeding, Sanya 572000, China; liuxiaochen1680@163.com (X.L.); liubo090325@163.com (B.L.); dyunpeng1997@gmail.com (Y.D.); 18889164038@163.com (W.M.); 994341@hainanu.edu.cn (W.W.); 2 Institute of Tropical Bioscience and Biotechnology, Chinese Academy of Tropical Agricultural Sciences, Haikou 570100, China; 3 Sanya Research Institute, Chinese Academy of Tropical Agricultural Sciences, Sanya 572000, China

**Keywords:** cassava, nitrate-deficiency, CC-type glutaredoxin, MeCEPD, nitrogen use efficiency

## Abstract

Cassava (*Manihot esculenta* Crantz) is a nitrogen-efficient crop that can achieve high biomass production on poor soils. However, the mechanisms underlying the response of cassava to nitrogen-deficiency signals and the regulation of nitrogen use efficiency remain unclear. Here, we found that *MeCEPD* (*MeGRXC1*) was specifically induced by CEP6 peptides and low nitrate, and showed higher expression in leaves and stems. Overexpression of *MeCEPD* enhanced cassava’s tolerance to nitrate deficiency by upregulating the expression of *MeNRT2.1*, *MeNRT2.4*, and *MeRBCS1A*, which was manifested as increased root biomass, greater lateral root number, and darker leaf coloration. In contrast, the *MeCEPD*-edited lines exhibited a statistically significant reduction in root length, plant height, and biomass compared to the wild-type. Additionally, nitrate deficiency accelerated leaf senescence. Furthermore, yeast two-hybrid (Y2H) assay revealed that MeCEPD interacts with the photosynthesis-related MeRBCS1A and lateral root development-related MeLHW, which may regulate nitrogen use efficiency. Unlike its *Arabidopsis thaliana* homologs AtCEPD1/2 and AtCEPDL2, which interact with AtTGA1/4, MeCEPD does not interact with MeTGA1 yet still upregulates *MeNRT2.1* expression. These findings contribute to our understanding of the complex regulatory mechanisms underlying cassava’s adaptation to low-nitrogen conditions and could provide new information for genetic improvement in nitrogen use efficiency in cassava.

## 1. Introduction

During the plant life cycle, both biotic and abiotic stressors can potentially disrupt cellular redox homeostasis by interfering with electron transfer. Glutaredoxins (GRXs) are small oxidoreductases of the thioredoxin superfamily that utilize reduced glutathione (GSH) as an electron donor. They catalyze protein disulfide bond reduction and glutathionylation/deglutathionylation reactions, thereby directly regulating the activity and function of target proteins. In plants, this redox regulatory mechanism enables GRXs to play a key role in maintaining cellular redox homeostasis and transducing stress and developmental signals [1].

GRXs are classified into four distinct subfamilies based on their active site sequences: CPYC-type (class I), CGFS-type (class II), CC-type, and GRL-type (class III) [2]. In addition, a GRL-type (GRX-like) with GRX functional characteristics but non-conserved active site motifs has been identified in *Arabidopsis thaliana* and rice (*Oryza sativa* L.) [3,4]. In *Arabidopsis*, CPYC-type GRXs possess the C[G/P/S]Y[C/S] active site motif and function primarily in maintaining cellular redox homeostasis [5,6]; CGFS-type GRXs contain the conserved CGFS motif and are involved in iron–sulfur cluster assembly. Both CPYC and CGFS types are present in prokaryotes and eukaryotes [7,8]. Notably, CC-type GRXs are unique to higher land plants, with active site motifs of CC[M/L][C/S/G] [9]. A total of 21 CC-type family members (AtROXY1-21) have been identified in *Arabidopsis*. Studies have shown that ROXY proteins play important roles in several key processes [10]. This includes floral organ development mediated by *AtROXY1* and *AtROXY2* [11], and plant defense responses to salicylic acid and jasmonate mediated by *AtGRX480* (*AtROXY19*) [12], among other documented functions.

Accumulating evidence indicates the critical role of CC-type GRX members in plant nitrogen nutrition signaling. Nitrate signals have been shown to induce the expression of *AtROXY11*, *AtROXY13*, and *AtROXY15*. Among them, overexpression of *AtROXY15* negatively regulates nitrate uptake and inhibits lateral root development [13,14,15]. Furthermore, studies have found that *AtROXY6* and *AtROXY9* are also involved in responding to nitrate signals, and their expression is downregulated [16]. Other research indicates that the expression of these two genes can be upregulated by CEP peptides, hence they are named *AtCEPD1* and *AtCEPD2* [17]. Intriguingly, when plants are exposed to heterogeneous nitrate distribution in soil, their nitrate-deficient neighboring lateral roots produce small CEP1 [18,19]. This peptide is then transported via the xylem to the shoot tissues, where it is perceived by the CEPR [20,21]. The resulting CEPD1/2 peptides are subsequently evenly distributed to both sides of the root via the phloem. This leads to a significant upregulation of *AtNRT2.1*, a high-affinity nitrate transporter, on the nitrate-rich side of the root.

Further investigations revealed that overexpression of *AtCEPDL2* (*AtROXY8*) in *Arabidopsis* significantly increased the expression of *AtNRT1.5*, *AtNRT2.4*, *AtNRT3.1*, and *AtNRT2.1*, suggesting a potential, yet undefined role in nitrate uptake and transport. *AtCEPDL2* is primarily and specifically expressed in leaves, with minimal expression in roots. Similar to AtCEPD1/2, AtCEPDL2 also acts as a mobile signaling molecule that can travel from the shoot to the root system. Grafting experiments have confirmed that *AtCEPDL2* can be directly induced by nitrate-starvation signals from the shoot, a process independent of the established CEP-CEPR-CEPD pathway [22].

In *Arabidopsis*, twenty ROXY proteins can interact with the bZIP transcription factor TGA2, playing roles in development and stress responses, and those that negatively regulate TGA2 activity share a conserved C-terminal ALWL motif [23,24]. Subsequent studies found that AtCEPD1/2 and AtCEPDL2, which lack the ALWL motif, can interact with AtTGA1 and its redundant homolog AtTGA4, with AtCEPD2 and AtCEPDL2 capable of interfering with TGA1 and TGA4 function [25]. TGA1/TGA4 can bind to the promoter regions of the *AtNRT2.1* and *AtNRT2.2* genes, regulating root morphogenesis and promoting nitrate uptake [26]. These findings demonstrate that the adaptation mechanisms plants employ to respond to low-nitrogen stress are far more complex than previously thought.

Cassava (*Manihot esculenta* Crantz) is a low-nitrogen-tolerant crop with high adaptability to heterogeneous nitrogen distribution in soil. Previous studies have shown that MeGRXC15 interacts with AtTGA5 and MeTGA074, and functions in drought tolerance through an ABA-dependent pathway [27]. MeGRXC3 interacts with AtTGA2 and AtTGA5 to modulate mannitol-induced osmotic stress response [28], and also interacts with MeTGA2 to regulate ROS homeostasis and stomatal movement under drought stress [29]. However, despite the well-documented role of CC-type GRXs in drought response, their function in nitrate deficiency remains unexplored. In this study, 19 CC-type GRX members were identified in cassava. Based on our previous finding that *MeCEP6* is strongly induced by low nitrate in cassava [30], we found CEP6 peptides can up-regulate *MeCEPD* expression, especially in leaves and stems. Functional characterization revealed that overexpression of *MeCEPD* conferred enhanced low-nitrate tolerance in plants including greater root biomass and nitrate accumulation, as well as more numerous and greener leaves. This was accompanied by upregulated expression of *MeNRT2.1*, *MeNRT2.4*, and *MeRBCS1A*. By contrast, *MeCEPD*-edited lines displayed suppressed root growth, reduced nitrate accumulation, and accelerated leaf senescence. Although lacking the ALWL motif, MeCEPD did not interact with MeTGA1 but instead regulated downstream pathways by interacting with the photosynthesis-related MeRBCS1A and the lateral root development-associated MeLHW protein. These findings reveal the molecular basis for efficient nitrogen use in cassava under nitrogen-limited conditions and provide new targets and strategies for improving nitrogen use efficiency in crops.

## 2. Results

### 2.1. Identification of CC-Type GRX Family in Cassava

**Figure 1:**
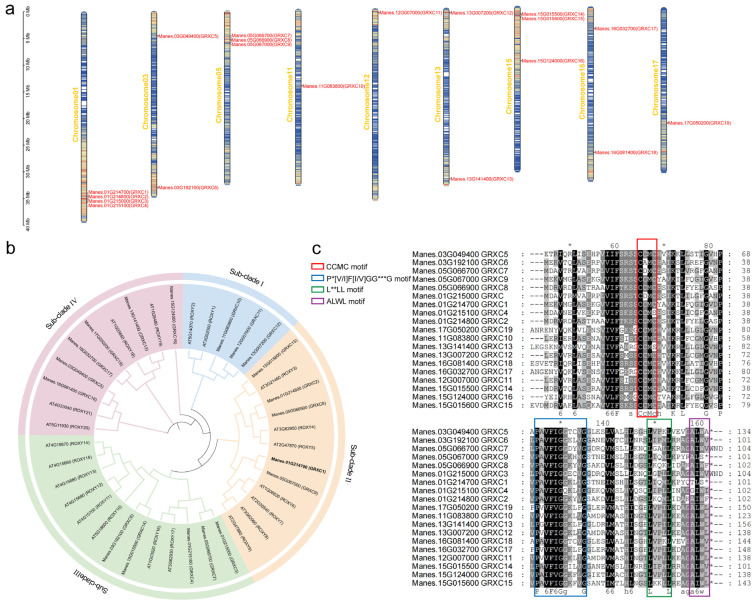
The chromosome distribution and phylogenetic tree analysis of cassava CC-type GRXs. (**a**) Chromosome localization of the *MeGRXC* gene family. (**b**) Homology comparison analysis of CC-type glutaredoxin-like proteins in *Arabidopsis* and cassava. A phylogenetic tree based on amino acid sequences was constructed using MEGA X (version 2.7) software with the neighbor-joining method. (**c**) Multiple sequence alignment of MeGRXC proteins. Conserved domains are indicated by colored boxes. Fully conserved residues are shaded in black, and partially conserved residues are shaded in gray. Dashes indicate gaps introduced to optimize alignment. “*” at the end of the sequences indicate stop codons. In the legend, “*” indicate non-conserved residues.

To identify CC-type GRX proteins in cassava, a hidden Markov model (HMM) was constructed based on the protein sequences of 21 *Arabidopsis thaliana* CC-type GRX genes (Supplementary Table S1). This model was used to screen the cassava protein database, and 19 CC-type GRX proteins were identified after manual verification of conserved domains. These 19 genes encode proteins ranging from 101 to 156 amino acids in length. The majority are intronless, with only a few containing 1 or 2 introns (Supplementary Table S2). The corresponding CC-type GRX genes in cassava were unevenly distributed across nine chromosomes, and were designated as *MeGRXC1*-*MeGRXC19* according to the chromosome localization (Figure 1a). Notably, three *MeGRXC* gene clusters were identified on chromosomes 1, 5, and 15, harboring *MeGRXC1-4*, *MeGRXC7-9*, and *MeGRXC14-15*, respectively, suggesting functional redundancy, expansion and potentially enhanced functionality of GRXs in cassava. Phylogenetically, *MeGRXC1*, *MeGRXC9*, *MeGRXC2*, *MeGRXC8*, and *MeGRXC15* (Sub-Clade II) clustered with *AtROXY6-9* implying a potential role in low-nitrate response (Figure 1b). The CC-type GRX functional sites, including the redox-active CCMC motif, the glutathione-binding P*[VI]F[IV]GG***G motif [28], and a C-terminal L**LL motif, are highly conserved between cassava and *Arabidopsis*. MeGRXC1 and MeGRXC9 share highly similar key amino acid sequences with AtROXY6-9 and both lack the C-terminal ALWL motif (Figure 1c), suggesting that this deletion may contribute to differential low-nitrate response within this subfamily.

### 2.2. The Expression Pattern of Cassava CC-Type Sub-Clade II MeGRXs

**Figure 2:**
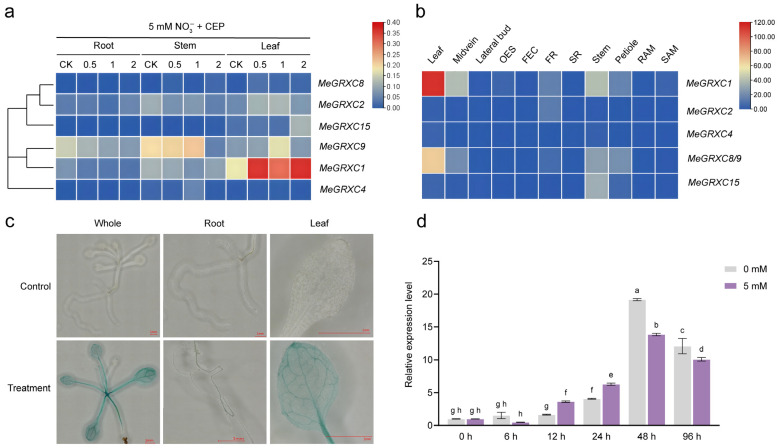
Expression analysis of cassava CC-type Sub-clade II *MeGRXC* genes. (**a**) Heatmap showing the expression levels of *MeGRXC* genes in roots, stems, and leaves of cassava seedlings treated with 5 mM NO_3_^−^ (CK), or 5 mM NO_3_^−^ supplemented with 0.5, 1, or 2 µM CEP6. (**b**) Tissue-specific expression profiles based on public transcriptome data. (**c**) GUS staining analysis of *MeCEPD* promoter activity in transgenic *Arabidopsis* seedlings. Plants expressing the *ProMeCEPD*::GUS fusion were grown under normal conditions and stained for GUS activity. Representative images show GUS signal in whole seedlings. Scale bar: 1 mm. (**d**) Time-course analysis of *MeCEPD* expression in cassava seedlings treated with 0 or 5 mM NO_3_^−^ for the indicated durations (0, 6, 12, 24, 48, and 96 h). Expression levels were measured by RT-qPCR. Data are presented as mean ± s.d. (*n* = 3). Different letters indicate statistically significant differences as determined by two-way ANOVA followed by Tukey’s post hoc test (*p* < 0.05).

Treatments with CEP6 at all tested concentrations (0.5, 1, and 2 µM) significantly upregulated the expression of *MeGRXC1*, *MeGRXC2*, and *MeGRXC9* in leaves (Figure 2a). Among them, *MeGRXC1* exhibited the highest induction level and was designated as *MeCEPD*. Notably, *MeGRXC4*, which belongs to a different phylogenetic branch from *MeCEPD* (Figure 1c), did not show significant expression changes in response to CEP6, indicating that members of this branch might not be sensitive to nitrate signals.

Analysis of cassava transcriptome data across tissues revealed that *MeCEPD* was highly expressed in leaves and stems, with detectable expression in leaf veins, somatic embryos, and petioles (Figure 2b). In contrast, *MeGRXC2* and *MeGRXC4* were weakly expressed in most tissues, although *MeGRXC2* showed relatively higher expression in adventitious roots. Histochemical staining of *MeCEPDpro*::GUS transgenic plants indicated strong promoter activity in leaves and stems, particularly within vascular bundles, with weaker activity in roots (Figure 2c).

Further investigation into the effect of nitrate concentration on *MeCEPD* expression revealed that *MeCEPD* expression was significantly upregulated after 24 h. At 48 h under 0 mM NO_3_^−^ conditions, *MeCEPD* expression reached its peak, significantly higher than that observed in the 5 mM NO_3_^−^ treatment. This indicates that *MeCEPD* is induced by nitrate deficiency stress.

### 2.3. Promoter Cis-Element Analysis and Protein Structure Prediction of MeCEPD

To investigate the potential transcriptional regulation of *MeCEPD*, a 2000 bp sequence upstream of its 5′ UTR was analyzed using the PlantCARE database. Multiple cis-acting regulatory elements involved in light responsiveness, hormone signaling, cell cycle control, and abiotic stress responses, including low nitrogen, drought, and low temperature, were identified in the *MeCEPD* promoter region (Table A1).

To further understand the regulatory features of *MeCEPD* among CC-type GRX family members, we performed a comparative analysis of cis-elements in the promoter regions of 19 cassava GRXC genes. We selected cis-elements related to phytohormone signaling (ABRE, CGTCA, TGACG, TCA, as-1) and stress responses (WRE3, TC-rich repeats, STRE, MBS, LTR, ARE), and visualized their distribution using TBtools II (version 2.441) (Supplementary Figure S1). This analysis revealed that MeCEPD is enriched in ABRE elements (cis-acting elements involved in ABA response), and also contains STRE, MBS, and LTR elements, but notably lacks MeJA-responsive (CGTCA, TGACG, as-1) and SA-responsive (TCA) elements, suggesting a distinct regulatory mechanism compared to other family members.

**Figure 3:**
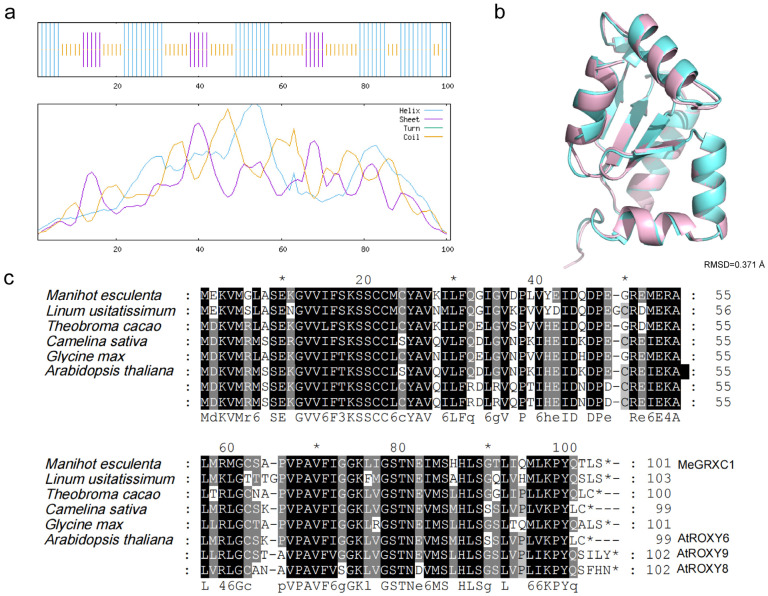
Promoter cis-element analysis and protein structure prediction of *MeCEPD.* (**a**) Predicted secondary structure of the MeCEPD protein using the SOPMA server. Helices, sheets, turns, and coils are represented by blue, red, green, and purple vertical lines, respectively. (**b**) Three-dimensional structural comparison of MeCEPD (blue) and *Arabidopsis* AtROXY9 (purple) generated by PyMOL software based on homology models. The RMSD value is 0.371 Å, indicating high structural similarity. (**c**) Multiple sequence alignment of MeCEPD with its putative orthologs from *Linum usitatissimum*, *Theobroma cacao*, *Glycine max*, *Camelina sativa*, and *Arabidopsis thaliana* (*AtROXY6*, *AtROXY8*, and *AtROXY9*). Fully conserved residues are shaded in black, and partially conserved residues are shaded in gray. Dashes indicate gaps introduced to optimize alignment. “*” at the end of the sequences indicate stop codons.

MeCEPD consists of 101 amino acid residues, with a predicted molecular weight of 11.01 kDa and an isoelectric point (pI) of 6.06, as determined using the ExPASy ProtParam tool. The secondary structure of the MeCEPD protein was predicted using the SOPMA server (Figure 3a). The predicted structure comprises 43.56% alpha helix, 14.85% extended strand, 41.58% random coil, and 0% beta turn. The three-dimensional structure of MeCEPD was predicted by homology modeling using SWISS-MODEL and compared with that of *Arabidopsis* AtROXY9 using PyMOL (version 2.5) software. Structural alignment revealed a high degree of similarity, with an RMSD value of 0.371 Å (Figure 3b), indicating strong structural conservation between these two CC-type glutaredoxins. Multiple sequence alignment of MeCEPD with its orthologs from *Linum usitatissimum*, *Theobroma cacao*, *Glycine max*, *Camelina sativa*, and *Arabidopsis thaliana* (AtROXY6, AtROXY8, and AtROXY9) revealed high evolutionary conservation of this CC-type glutaredoxin (Figure 3c).

### 2.4. MeCEPD Localized in the Nucleus and Cytoplasm

**Figure 4:**
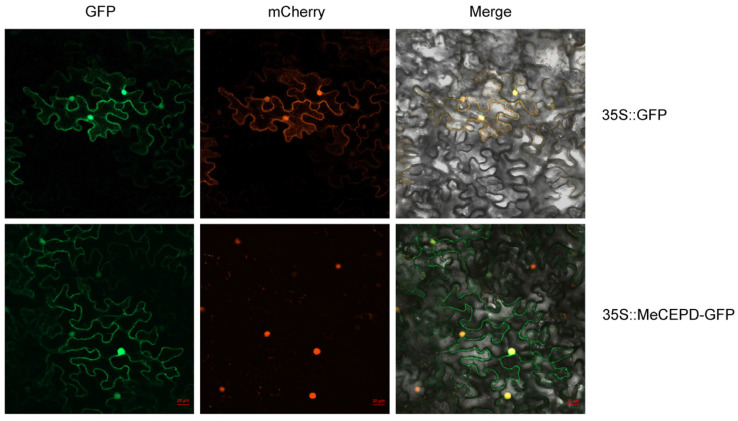
Subcellular localization of MeCEPD in *Nicotiana benthamiana* leaves. (**Upper panels**) Free GFP control was co-expressed with a plasma membrane marker and a nuclear marker. GFP fluorescence was observed in the cytoplasm, nucleus, and plasma membrane. (**Lower panels**) The MeCEPD-GFP fusion protein was co-expressed with the nuclear marker (mCherry). GFP signal was detected in both the cytoplasm and nucleus. Merged images show overlay of GFP, mCherry, and bright-field channels. Scale bars: 20 μm.

To determine the subcellular localization of MeCEPD, the *MeCEPD*-GFP fusion construct was transiently expressed in tobacco (*Nicotiana benthamiana*) leaves. Confocal microscopy revealed that the MeCEPD-GFP fusion protein localized to both the cytoplasm and nucleus. In contrast, the free GFP control exhibited fluorescence throughout the entire cell, including the cytoplasm, nucleus, and plasma membrane (Figure 4). These results indicate that MeCEPD is a protein with both cytoplasmic and nuclear localization.

### 2.5. MeCEPD Enhances Nitrate Deficiency Tolerance in Cassava

**Figure 5:**
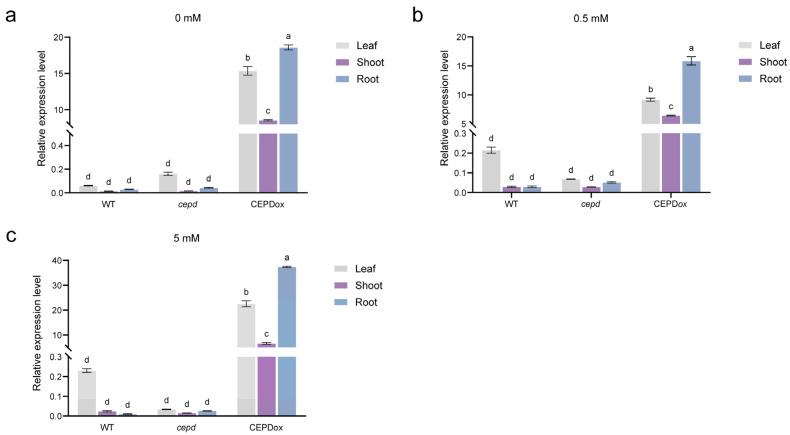
Expression analysis of *MeCEPD* in transgenic cassava plants under different nitrate concentrations. (**a**–**c**) RT-qPCR analysis of *MeCEPD* transcript levels in roots, stems, and leaves of wild-type (WT), *cepd*-edited (*cepd*), and *MeCEPD*-overexpressing (CEPDox) cassava plants treated with 0 mM (**a**), 0.5 mM (**b**), or 5 mM (**c**) NO_3_^−^ for 30 days. Data are presented as mean ± s.d. from three biological replicates. Data were analyzed by two-way ANOVA with genotype, tissue, and treatment as factors, followed by Tukey’s post hoc test. Different letters indicate statistically significant differences among genotypes within each tissue and treatment (*p* < 0.05).

We first analyzed *MeCEPD* expression across different tissues and nitrate conditions. RT-qPCR revealed that under nitrate deprivation (0 mM), *MeCEPD* transcript levels showed no significant difference between wild-type (WT) and *cepd*-edited lines (*cepd*). In *MeCEPD*-overexpressing (CEPDox) lines, however, *MeCEPD* expression was highest in roots, intermediate in leaves, and lowest in stems. This root-predominant expression pattern was consistently observed under both 0.5 mM and 5 mM nitrate treatments, confirming that *MeCEPD* overexpression significantly elevates its transcript levels in roots (Figure 5).

**Figure 6:**
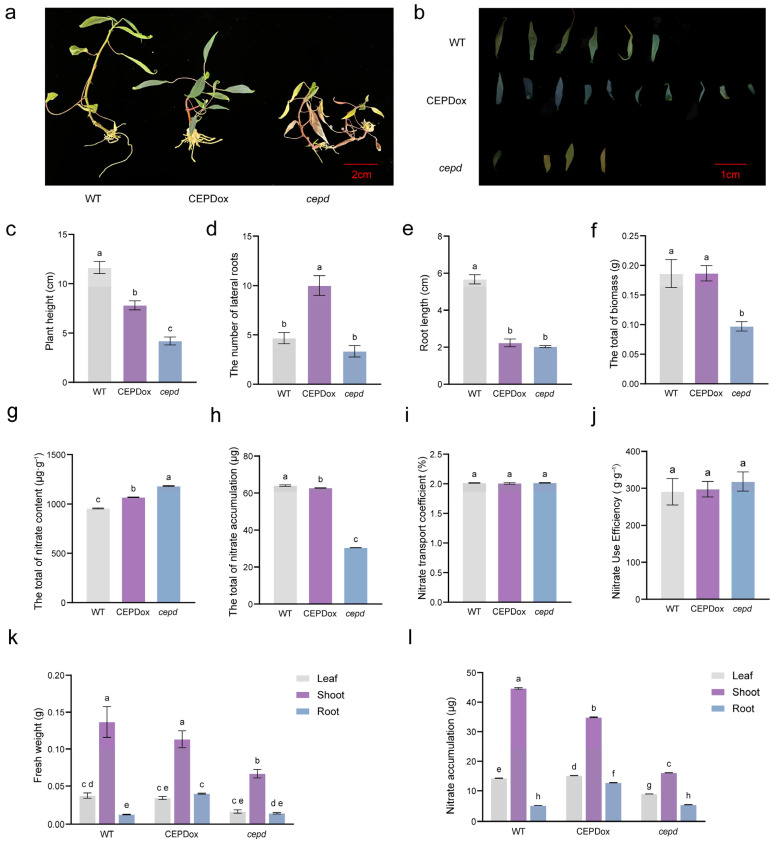
Physiological characterization of *MeCEPD* transgenic cassava plants under 0 mM NO_3_^−^ conditions. (**a**,**b**) Phenotypes of WT, *cepd*, and CEPDox plants after 35 days of liquid culture with 0 mM NO_3_^−^. (**a**) Whole-plant morphology. Scale bar: 2 cm. (**b**) Leaf phenotypes. Scale bar: 1 cm. (**c**) Plant height. (**d**) Number of lateral roots. (**e**) Root length. (**f**) Total biomass. (**g**) Nitrate content. (**h**) Nitrate accumulation. (**i**) Nitrate translocation coefficient. (**j**) Nitrate use efficiency. For (**c**–**j**), different letters indicate statistically significant differences among genotypes as determined by one-way ANOVA followed by Tukey’s post hoc test (*p* < 0.05). (**k**) Biomass of roots, stems, and leaves. (**l**) Nitrate accumulation in roots, stems, and leaves. Data are presented as mean ± s.d. For (**k**,**l**), data were analyzed by two-way ANOVA with genotype and tissue as factors, followed by Tukey’s post hoc test. Different letters indicate statistically significant differences (*p* < 0.05).

To further investigate the functional role of *MeCEPD* in cassava, we evaluated a range of growth and physiological parameters in WT, *cepd*, and CEPDox plants under 0 mM NO_3_^−^ treatment. Under 0 mM NO_3_^−^ conditions, *cepd* plants exhibited severe stress symptoms (Figure 6a,b), including stunted growth, leaf yellowing and wilting, reduced plant height and root length, and significantly decreased total biomass. In contrast, CEPDox plants displayed enhanced stress tolerance. Although their plant height was slightly lower than that of WT plants, the CEPDox maintained greener leaves, developed significantly more lateral roots, and showed markedly higher root biomass and root nitrate accumulation than WT. Notably, both total biomass and nitrate accumulation in CEPDox plants were comparable to those in WT, whereas *cepd* plants exhibited the highest nitrate content yet the lowest total nitrate accumulation. No significant differences were observed among genotypes in nitrate root-to-shoot translocation coefficient or nitrate use efficiency (Figure 6c–l). Together, these results indicate that *MeCEPD* contributes to cassava tolerance to nitrate deficiency conditions by promoting root development and facilitating internal nitrate remobilization.

### 2.6. MeCEPD Is Involved in Regulation of Key Genes in Nitrate Uptake and Assimilation Under Nitrate Deficiency

**Figure 7:**
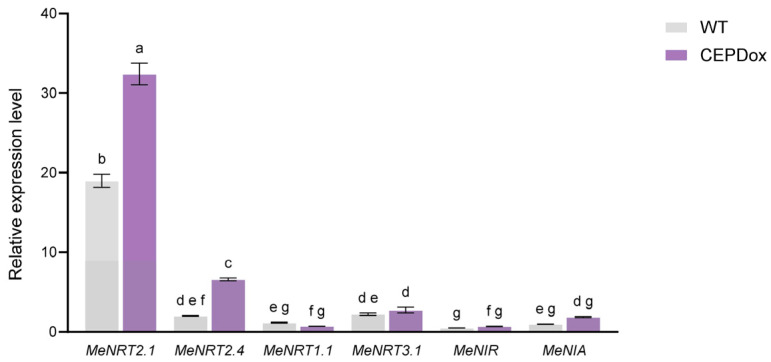
*MeCEPD* overexpression upregulates key nitrate uptake and assimilation genes in roots under 0 mM NO_3_^−^ conditions. RT-qPCR analysis of nitrate transporter and reductase genes in WT and CEPDox plants subjected to 0 mM NO_3_^−^ for 35 days. Values are presented as mean ± s.d. (*n* = 3). Data were analyzed by two-way ANOVA followed by Tukey’s post hoc test for multiple comparisons. Different letters indicate statistically significant differences among genotypes for each gene (*p* < 0.05).

To investigate whether *MeCEPD* is involved in nitrate uptake and assimilation, we analyzed the expression of key transporter and reductase genes including *MeNRT1.1*, *MeNRT2.1*, *MeNRT2.4*, *MeNRT3.1*, *MeNIA*, and *MeNIR* in roots of WT and CEPDox plants under 0 mM NO_3_^−^ treatment. RT-qPCR analysis revealed that *MeNRT2.1* was the most highly induced gene under this nitrate-deficiency condition. Among the genes tested, only *MeNRT2.1* and *MeNRT2.4* showed significant upregulation in CEPDox lines compared to WT, while no significant differences were observed for the other genes (Figure 7). These results indicate that *MeCEPD* activates the expression of critical genes involved in nitrate transport, suggesting its role in enhancing nitrate uptake and assimilation in roots under nitrate deficiency.

### 2.7. MeCEPD Interacts with and Upregulates MeRBCS1A in Response to Nitrate Deficiency

**Figure 8:**
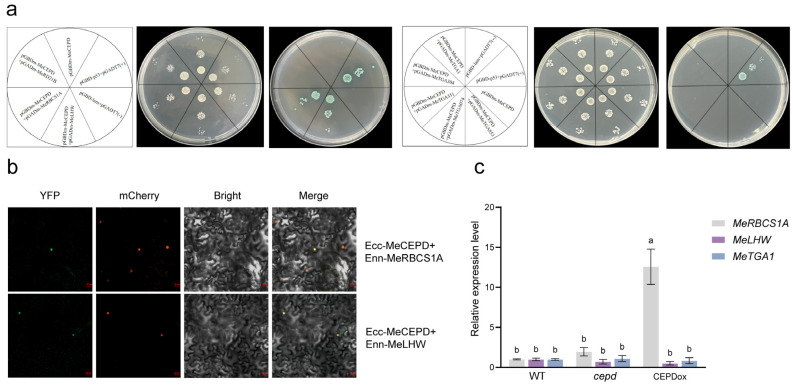
MeCEPD interacts with MeRBCS1A and MeLHW and upregulates *MeRBCS1A* expression under nitrate deficiency. (**a**) Yeast two-hybrid assays showing that MeCEPD interacts with MeRBCS1A and MeLHW, but not with TGA transcription factors. (**b**) BiFC assays validating the interaction of MeCEPD with MeRBCS1A and MeLHW in the nucleus of *N. benthamiana* leaf cells. Scale bars: 20 μm. (**c**) RT-qPCR analysis of *MeRBCS1A*, *MeLHW*, and *MeTGA1* expression in roots of WT, *cepd*, and CEPDox plants under 0 mM NO_3_^−^ conditions for 35 days. Expression levels are shown relative to WT. Data are presented as mean ± s.d. (*n* = 3). Data were analyzed by two-way ANOVA with genotype and gene as factors, followed by Tukey’s post hoc test for multiple comparisons. Different letters indicate statistically significant differences (*p* < 0.05).

In *Arabidopsis*, CC-type glutaredoxins have been reported to interact with TGA transcription factors to regulate development and defense responses. Whether MeCEPD interacts with TGA proteins in cassava remained unknown. To address this, we performed yeast two-hybrid assays using MeCEPD as bait. Several cassava TGA homologs, including TGA1, TGA304, TGA351, TGA074, and TGA853, were tested, but none showed interaction with MeCEPD (Figure 8a).

To identify potential interacting partners of MeCEPD under nitrate-deficient conditions, we screened a nitrogen-treated fibrous root cDNA library by yeast two-hybrid assay. Among 19 candidate proteins initially identified, a cysteine protease MeBD21B, a Rubisco small subunit MeRBCS1A, and a transcription factor MeLHW were selected for pairwise validation. Only MeRBCS1A and MeLHW were confirmed to interact with MeCEPD in yeast (Figure 8a). Bimolecular fluorescence complementation (BiFC) assays further validated that MeCEPD interacts with MeRBCS1A and MeLHW in the nucleus of plant cells (Figure 8b).

To investigate whether MeCEPD regulates the expression of its interacting proteins under nitrate deficiency, we examined the transcript levels of *MeRBCS1A*, *MeLHW*, and *MeTGA1* in WT, *cepd*, and CEPDox lines under 0 mM NO_3_^−^ conditions. RT-qPCR analysis revealed that *MeRBCS1A* expression was significantly upregulated in CEPDox plants (approximately 13-fold, *p* < 0.05) compared with WT under nitrate deficiency (Figure 8c). In contrast, no significant changes were observed in the expression of *MeLHW* or *MeTGA1*. These results indicate that MeCEPD specifically upregulates *MeRBCS1A* expression under nitrate deficiency, suggesting that MeRBCS1A may function as a downstream effector of MeCEPD in mediating cassava responses to nitrate deficiency.

## 3. Discussion

### 3.1. MeCEPD Enhances Cassava Adaptation to Nitrate Deficiency by Upregulating MeNRT2.1 Expression

Under low-nitrate conditions, plants enhance nitrate acquisition by regulating the expression of high-affinity nitrate transporter genes. In *Arabidopsis*, rice, and other plant species, *NRT2.1* responds to low-nitrate signals and plays a critical role in nitrate uptake and biomass accumulation [31,32]. The expression of cassava *MeNRT2.1* and *MeNRT2.2* is also induced by low nitrate [33], and heterologous expression of *MeNRT2.1* promotes nitrate uptake in *Arabidopsis* roots while increasing chlorophyll and nitrate reductase content in leaves [34]. In *Arabidopsis*, the CC-type glutaredoxins *AtCEPD1/2* and *AtCEPDL2* are upregulated under low-nitrate conditions, and their overexpression significantly enhances the transcriptional activity of *AtNRT2.1* [16,22].

In this study, we identified *MeGRXC1* as a cassava homolog closely related to *Arabidopsis CEPDs* and confirmed that its expression is induced by CEP6 and nitrate deficiency (Figure 2); accordingly, it was designated *MeCEPD*. To investigate the adaptation mechanisms of cassava to nitrate deficiency, we employed complete nitrate deprivation (0 mM NO_3_^−^) in this study. Functional analysis revealed that under 0 mM NO_3_^−^ conditions, overexpression of *MeCEPD* significantly upregulated the expression of *MeNRT2.1* and *MeNRT2.4* (Figure 7). Compared with wild-type (WT) plants, *MeCEPD*-overexpressing (CEPDox) lines exhibited darker green leaves and increased root biomass, whereas *cepd* mutants displayed reduced plant size, suppressed root development, and decreased total nitrate accumulation (Figure 6).

Interestingly, although *cepd* mutants showed the highest nitrate content per unit mass, their total nitrate accumulation was the lowest (Figure 6g,h). This inverse relationship between nitrate content and biomass in the *cepd* line reflects a concentration effect resulting from severe growth inhibition, rather than enhanced nitrate retention capacity.

No significant differences were observed between CEPDox and WT plants in total biomass, nitrate translocation coefficient, or nitrate use efficiency (Figure 6). This may be attributed to extreme nitrate deficiency (0 mM NO_3_^−^), under which plants are unable to take up sufficient nitrogen from the external medium even with significantly higher expression levels of *MeNRT2.1* and *MeNRT2.4*, thereby failing to support growth and biomass accumulation. Alternatively, due to prolonged nitrate starvation (35 days), plant growth becomes limited by carbon metabolism, and the early advantages conferred by *MeCEPD* overexpression diminish over time. However, CEPDox plants accumulated significantly higher nitrate levels in roots compared to WT (Figure 6l), suggesting that under extreme nitrate deficiency, the adaptive strategy prioritizes maintaining root architecture and local nitrate remobilization over enhancing overall nitrate uptake via *MeNRT2.1*.

Nitrogen deficiency is known to induce reactive oxygen species (ROS) accumulation, which contributes to leaf senescence and chlorophyll degradation [35,36]. The accelerated leaf yellowing observed in *cepd* mutants under 0 mM NO_3_^−^ conditions (Figure 6b) raises the possibility that *MeCEPD* may be involved in ROS homeostasis. In cassava, another CC-type glutaredoxin, MeGRXC3, has been demonstrated to regulate H_2_O_2_ distribution through modulation of catalase activity [29]. Given that MeCEPD belongs to the same CC-type GRX subfamily, it may similarly participate in maintaining redox balance under nitrate deficiency. Future studies measuring ROS levels and antioxidant enzyme activities in *cepd* and CEPDox lines would help clarify whether *MeCEPD* plays a role in ROS regulation during nitrate starvation. Furthermore, the functional relationship between *MeCEPD* and *MeNRT2.1* in nitrate response warrants further investigation under low-nitrate conditions, and whether *MeNRT2.1* serves as a direct downstream target of MeCEPD remains to be experimentally validated.

### 3.2. MeCEPD-Mediated Regulation of MeNRT2.1 Is Independent of TGA Transcription Factors

CC-type glutaredoxins (GRXs) typically interact with TGA transcription factors to co-regulate plant growth and developmental processes [37]. In *Arabidopsis*, TGA transcription factors are classified into five groups based on sequence similarity: Group I (TGA1/4), Group II (TGA2/5/6), Group III (TGA3/7), Group IV (TGA9/10), and Group V (PAN) [38]. Previous studies have demonstrated that *Arabidopsis* ROXY19 interacts with Group II TGA5/6 to participate in detoxification pathways [39]; ROXY1/2 interact with Group IV TGA9/10 to regulate anther development [40]; and ROXY1 interacts with Group V PAN to control petal number [41]. Furthermore, under low-nitrate conditions, AtCEPD1/2 and AtCEPDL2, which lack the ALWL motif, interact with Group I TGA1/4 in the nucleus and interfere with the inhibitory effect of TGA1/4 on *AtNRT2.1* and *AtNRT2.2*, thereby coordinately regulating root architecture and enhancing nitrate uptake and assimilation [25,26].

In cassava, CC-type glutaredoxins such as MeGRXC3 and MeGRXC15 have been shown to interact with TGA transcription factors to regulate drought tolerance [27,28,29]. However, in this study, we found that MeCEPD shares high sequence similarity with *Arabidopsis* AtCEPD1/2 and AtCEPDL2, but unlike these homologs, yeast two-hybrid assays revealed that MeCEPD does not interact with MeTGA1, nor with other MeTGA family members tested MeTGA074 (Group II), MeTGA853 (Group III), MeTGA304 (Group IV), and MeTGA351 (Group V). Further experiments, such as BiFC, LCI, and Co-IP, are needed to definitively determine whether MeCEPD interacts with MeTGA1. Taken together, cassava MeCEPD may regulate *MeNRT2.1* expression through a novel transcription factor that differs from *Arabidopsis*.

### 3.3. MeCEPD Interacts with MeRBCS1A and MeLHW to Regulate Carbon–Nitrogen Balance and Root Development

To identify MeCEPD-interacting proteins, we performed a yeast two-hybrid screen of a cassava cDNA library. No TGA family members were identified among the candidate interactors. From the candidate pool, MeRBCS1A and MeLHW were confirmed to interact with MeCEPD by yeast two-hybrid and BiFC assays (Figure 8a,b).

Nitrogen is a constituent of chlorophyll, and nitrogen deficiency reduces the activity of photosynthesis-related enzymes. Rubisco, which accounts for 50% of soluble protein in leaf cells, is a key enzyme determining carbon assimilation rate in photosynthesis and also plays a critical role in photorespiration; its activity directly affects carbon–nitrogen metabolic balance [42]. *RBCS* encodes the small subunit of Rubisco. In *Arabidopsis*, double mutation of *AtRBCS1A* and *AtRBCS3B* reduces leaf Rubisco content, chlorophyll levels, soluble protein content, and nitrogen content [43]. In this study, we found that under 0 mM NO_3_^−^ conditions, *MeRBCS1A* expression was significantly upregulated in *MeCEPD*-overexpressing lines (Figure 8c), suggesting that MeCEPD may maintain photosynthetic carbon metabolism under nitrogen deficiency by influencing Rubisco assembly or stability.

In *Arabidopsis*, the AtHY5 transcription factor specifically binds to the GATA motif in the *AtRBCS1A* promoter [44]. Notably, HY5 also responds to low-nitrate signals and activates *NRT2.1* expression, thereby promoting root growth and nitrate uptake [45,46]. Both AtHY5 and AtCEPD are mobile proteins that can be transported long distances from shoots to roots via the phloem; AtCEPD responds to low-nitrate signals and activates *AtNRT2.1* expression in roots. Based on these findings, we hypothesize that MeRBCS1A may coordinately function with MeHY5 and MeCEPD to regulate carbon–nitrogen balance under nitrogen-deficient conditions in cassava—a hypothesis that warrants further experimental validation.

In *Arabidopsis*, LHW is primarily involved in vascular development and root architecture formation [47]. The interaction between MeCEPD and MeLHW may contribute to lateral root development, providing a plausible explanation for the significantly increased lateral root number observed in *MeCEPD*-overexpressing lines (Figure 6d). Transcript levels of *MeLHW* were unchanged in overexpression lines (Figure 8c), suggesting that MeCEPD may regulate MeLHW activity through post-translational modifications rather than transcriptional regulation.

## 4. Materials and Methods

### 4.1. Plant Materials and Growth Conditions

Cassava (SC8) plants were maintained as sterile tissue cultures in a growth room with a 12-h light/12-h dark photoperiod, light intensity of 250 μmol·m^−2^·s^−1^, and temperature of 28 ± 2 °C.

*Nicotiana benthamiana* plants were grown in a greenhouse for transient expression assays, with a 12-h light/12-h dark photoperiod, light intensity of 200 μmol·m^−2^·s^−1^, and temperature of 25 ± 2 °C.

*Arabidopsis thaliana* (Col-0) plants were grown in a separate growth room with a 14-h light/10-h dark photoperiod, light intensity of 150 μmol·m^−2^·s^−1^, and temperature of 22 ± 2 °C.

### 4.2. Cassava Nitrate Treatments

CEP6 peptide treatment: The mature CEP6 peptide (sequence: GWMPDGSVPSPGVGH) was synthesized by Sangon Biotech (Shanghai, China) as described in our previous study [30]. Cassava SC8 seedlings were cultured on 1/2 MS solid medium (Duchefa, M0222 (Haarlem, The Netherlands)) for 15 days and then transferred to four liquid-culture treatments: nitrogen-free MS liquid medium (PhytoTech Labs, M531 (Lenexa, KS, USA)) containing 5 mM NO_3_^−^ (as NaNO_3_), 5 mM NO_3_^−^ + 0.5 μM CEP6, 5 mM NO_3_^−^ + 1 μM CEP6, or 5 mM NO_3_^−^ + 2 μM CEP6, and further cultured for an additional 20 days. For each treatment, three seedlings were used as individual biological replicates (*n* = 3). The root, stem, and leaf samples were collected rapidly, frozen in liquid nitrogen and stored at −80 °C.

Nitrate Treatment at Different Time Points: 15-day-old SC8 seedlings were transferred from 1/2 MS solid medium to nitrogen-free MS liquid medium supplemented with either 0 mM or 5 mM NO_3_^−^. Root, stem, and leaf samples were collected at 0, 6, 12, 24, 48, and 96 h after transfer. At each time point, three seedlings were harvested as individual biological replicates (*n* = 3).

Treatment of Transgenic Plants: Wild-type (WT) and *MeCEPD* transgenic cassava plants (*cepd* and CEPDox) were initially cultured on 1/2 MS medium for 30 days. Subsequently, they were transferred to and maintained in nitrogen-free MS liquid medium containing 0 mM, 0.5 mM, or 5 mM NO_3_^−^ for an additional 35 days. The culture medium was not renewed during this period. Three plants per genotype were used as biological replicates for phenotypic measurements and gene expression analysis (*n* = 3). Following the treatment, root, stem, and leaf samples were collected and immediately frozen at −80 °C for storage.

### 4.3. Public Transcriptome Data

For tissue-specific expression analysis (Figure 2b), transcriptome data were obtained from the cassava gene expression atlas published by Wilson et al. [48] (2017, GEO accession: GSE82279). This dataset includes expression profiles for 11 cassava tissue/organ types: leaf, leaf midvein, petiole, stem, lateral bud, shoot apical meristem (SAM), storage root (SR), fibrous root (FR), root apical meristem (RAM), organized embryogenic structures (OES), and friable embryogenic callus (FEC). FPKM values were retrieved and used to generate the heatmap.

### 4.4. Identification of CC-Type GRXs (MeGRXCs) in Cassava

Based on the PFAM protein domain database (http://pfam-legacy.xfam.org/; accessed on 15 May 2022), a hidden Markov model (HMM) for CC-type GRXs was constructed. This model was used to screen the cassava AM560 genome (https://phytozome.jgi.doe.gov; accessed on 20 May 2022) to identify candidate genes with similar structures for subsequent analysis. A phylogenetic tree of CC-type GRXs from *Arabidopsis* and cassava was constructed using MEGA X (version 10.2) software and visualized and annotated with the iTOL online tool (https://itol.embl.de/; accessed on 7 January 2023). Furthermore, protein sequences were aligned using GeneDoc (version 2.7) software, and conserved motifs within the amino acid sequences of MeGRXCs were identified using the MEME Suite (https://meme-suite.org/meme/doc/meme.html; accessed on 21 February 2023).

### 4.5. Protein Structure Prediction and Promoter Analysis of MeCEPD

The physicochemical properties of the MeCEPD protein, including molecular weight and isoelectric point (pI), were predicted using the ExPASy ProtParam tool (https://web.expasy.org/protparam/; accessed on 2 March 2023). The secondary structure of the MeCEPD protein was predicted based on its amino acid sequence using the online tool SOPMA (https://npsa.lyon.inserm.fr/cgi-bin/npsa_automat.pl?page=/NPSA/npsa_sopma.html; accessed on 2 March 2023). A three-dimensional model of MeCEPD was generated by homology modeling using the SWISS-MODEL server (https://swissmodel.expasy.org/interactive; accessed on 2 March 2023). Structural comparison between MeCEPD and *Arabidopsis* AtROXY9 was performed using PyMOL (version 2.5) software (Schrödinger, LLC (New York, NY, USA)). The structures were aligned using the align command, and the root-mean-square deviation (RMSD) value was calculated to assess structural similarity. To analyze the promoter region, a 2000 bp sequence upstream of the 5’UTR of *MeCEPD* was retrieved from the cassava genome database. Putative cis-acting elements within this promoter sequence were subsequently predicted using the online tool PlantCARE (http://bioinformatics.psb.ugent.be/webtools/plantcare/html/; renewed on 7 March 2026). For comparative analysis, promoter sequences of 19 cassava GRXC genes were analyzed using PlantCARE, and the distribution of cis-elements was visualized using TBtools II (version 2.441).

### 4.6. GUS Staining

The promoter sequence of *MeCEPD* was cloned into the GUS reporter vector pCAMBIA-1381Z. The resulting recombinant plasmid was introduced into *Agrobacterium tumefaciens* strain GV3101 and then transformed into *Arabidopsis thaliana* via the floral dip method. GUS activity was detected histochemically using X-Glucuronide (X-Gluc) as the substrate, and the staining patterns were observed under a light microscope.

### 4.7. Creation of MeCEPD Transgenic Plants

*MeCEPD* transgenic cassava plants were generated via *Agrobacterium tumefaciens* (strain LBA4404)-mediated transformation of embryogenic callus (cultivar SC8), following the methodology described by Zhang et al. [49].

For gene editing, a single guide RNA (sgRNA) targeting the coding region of *MeCEPD* was designed and cloned into the pCAMPIA1301-Cas9 vector (Supplementary Figure S2a). Five independent CRISPR/Cas9-edited lines were obtained and verified by Hi-TOM sequencing (Supplementary Table S3). Among these lines, line #2 carries a homozygous 2-bp deletion in the coding region, resulting in a frameshift and premature stop codon, thereby generating a loss-of-function knockout mutant. This line (designated *cepd*) was selected for phenotypic analysis.

For overexpression, the full-length coding sequence of *MeCEPD* was cloned into the pCAMBIA1300-GFP vector under the control of the CaMV 35S promoter. Seven independent overexpression lines were generated, and *MeCEPD* transcript levels were examined by qPCR analysis (Supplementary Figure S2b). Overexpression line #2 (designated CEPDox) was selected for phenotypic analysis. The wild-type (WT) plants used in this study were subjected to the same transformation and selection procedure as the transgenic lines, but without stable integration of the transgene.

### 4.8. RT-qPCR Analysis

Total RNA was extracted from cassava leaves, stems, and roots using the RNAprep Pure Plant Kit (TIANGEN (Beijing, China)). First-strand cDNA was synthesized with the FastQuant RT Kit (Novazyme (Nanjing, China)). Quantitative PCR was performed using MonAmp^TM^ SYBR^®^ Green qPCR Mix (Monad (New York, NY, USA)) on a LightCycler^®^ 96 Real-Time PCR System (Roche (Basel, Switzerland)). Gene-specific primers (Supplementary Table S4) were used. Transcript levels were quantified using the comparative 2^−ΔΔCt^ method.

### 4.9. Phenotypic Characterization

Phenotypic images were captured for wild-type (WT), *MeCEPD*-overexpressing (CEPDox), and *MeCEPD*-edited (*cepd*) cassava plants. Plant height was measured, and the fresh weight (g) of roots, stems, and leaves was recorded separately. Total root length and lateral root number were analyzed using a root scanner. Nitrate (NO_3_^−^) content in roots, stems, and leaves was determined using a nitrate content assay kit (Cominbio, ZXTD-1-G (Suzhou, China)) following the manufacturer’s instructions, and expressed as μg·g^−1^ FW. Based on the measured data, the following parameters were calculated:

Nitrate translocation coefficient (%) = Nitrate content in aboveground parts/Nitrate content in underground parts × 100%

Total nitrate accumulation (μg) = Nitrate content (μg·g^−1^ FW) × Plant fresh weight (g)

Nitrate use efficiency (g.g^−1^) = Plant fresh weight (g)/Total nitrate accumulation (g)

(Note: For nitrate use efficiency calculation, total nitrate accumulation should be converted from μg to g by dividing by 10^6^).

### 4.10. Yeast Two-Hybrid Assay

A yeast two-hybrid screen was performed using the Matchmaker Gold Yeast Two-Hybrid System. The *MeCEPD* gene was cloned into the pGBDm vector (bait construct, a derivative of pGBKT7 with a modified multiple cloning site) and used to screen a cassava fibrous-root cDNA library.

The cDNA library was constructed by Oebiotech (Shanghai, China) using the Gateway system, from 242 root samples representing diverse cassava varieties, nitrogen treatments, and culture conditions. Root samples were collected from:

(1) Short-term hydroponic treatments: Seven cassava varieties (AM560, BRA755, C3, SC8, SC205, FB6, 18R) were cultured on MS medium for 22 days, transferred to nitrogen-free MS medium for 3 days of nitrogen starvation, and then subjected to homogeneous treatments (-N, NH_4_^+^, NO_3_^−^) and split-root treatments (-N/NH_4_^+^, -N/NO_3_^−^, NH_4_^+^/NO_3_^−^). Root samples were collected at 0 h, 6 h, and 72 h during nitrogen starvation, and at 2 h, 8 h, and 48 h after treatment initiation.
(2) Long-term pot treatments: Four cassava varieties (CH16, SC16, SC205, 17Q) were grown in 1/4-strength Hoagland solution with modified nitrogen sources under the same homogeneous and split-root treatments described above. Root samples were collected 24 days after treatment.

All root samples were divided into 10 pools based on treatment type and sampling time for RNA extraction and library construction.

Nineteen putative interacting proteins were initially identified. For interaction validation, the cDNA sequences of candidate proteins (MeRBCS1A, MeLHW, and MeTGAs) were cloned into pGADm (prey vector, a derivative of pGADT7 with a modified multiple cloning site). Each candidate construct was pair-wise co-transformed with the *MeCEPD*-pGBKm bait into the Y2HGold yeast strain. Transformants were selected on SD/-Trp/-Leu (DDO) plates, and protein–protein interactions were confirmed by β-galactosidase activity assay according to the manufacturer’s protocol.

### 4.11. Bimolecular Fluorescence Complementation (BiFC) Assay

To further verify the interactions between MeCEPD and MeRBCS1A or MeLHW, BiFC assays were carried out in *Nicotiana benthamiana* leaves via transient expression. The full-length coding sequences (without stop codons) of *MeCEPD*, *MeRBCS1A*, and *MeLHW* were separately fused to the N-terminal (Enn) or C-terminal (Ecc) fragment of yellow fluorescent protein (YFP) in the pBiFC vectors, generating 35S:*MeCEPD*-Enc, 35S:*MeRBCS1A*-Enn, and 35S:*MeLHW*-Enn constructs. These plasmids were introduced into *Agrobacterium tumefaciens* strain GV3101 (pSoup) and co-infiltrated in pairwise combinations into tobacco leaf epidermal cells. YFP fluorescence was observed 2–3 days after infiltration using a confocal microscope (ZEISS LSM 800) with appropriate YFP/GFP filter sets.

### 4.12. Protein Subcellular Localization

The *MeCEPD* coding sequence (without the stop codon) was amplified and subsequently fused in-frame to the N-terminus of GFP, then cloned downstream of the CaMV 35S promoter in the PSL 1 plus vector (a derivative of pCAMBIA1301 with a modified multiple cloning site) to generate the 35S::*MeCEPD*-GFP construct. This construct and the empty PSL 1 plus vector (35S::GFP) were introduced into *Agrobacterium tumefaciens* strain GV3101 and transiently expressed in *Nicotiana benthamiana* leaves via agroinfiltration. For subcellular localization analysis, the plasma membrane marker and nuclear marker were co-expressed with the free GFP control, while the nuclear marker mCherry was co-expressed with the MeCEPD-GFP fusion protein. GFP fluorescence was detected 2–3 days post-infiltration using a laser-scanning confocal microscope (ZEISS LSM 800).

### 4.13. Statistical Analysis

All data were presented as mean ± standard deviation (s.d.) from at least three independent biological replicates. Statistical analyses were performed using SPSS (version 19.0) or GraphPad Prism (version 10.6). For comparisons among three or more groups, one-way analysis of variance (ANOVA) was used, followed by Tukey’s post hoc test for pairwise multiple comparisons. For two-factor experimental designs, two-way ANOVA was used, followed by Tukey’s post hoc test to assess main effects and interactions. Different letters above bars indicate statistically significant differences at *p* < 0.05 after post hoc tests.

## 5. Conclusions

This study reveals MeCEPD as a key CC-type glutaredoxin mediating cassava adaptation to nitrate starvation. *MeCEPD* expression is specifically induced by CEP6 peptides and nitrate deficiency, with predominant expression in leaves and stems. Functional analyses demonstrate that *MeCEPD* overexpression enhances nitrate transporter gene expression (*MeNRT2.1*, *MeNRT2.4*), promotes lateral root development, and increases root nitrate accumulation under 0 mM NO_3_^−^ conditions. In contrast, *cepd* mutants exhibit severe growth inhibition and accelerated leaf senescence.

Importantly, MeCEPD operates independently of the TGA transcription factor pathway—a divergence from its *Arabidopsis* homologs AtCEPD1/2 and AtCEPDL2—yet retains the ability to upregulate *MeNRT2.1* expression. Through direct interactions with the photosynthesis-related protein MeRBCS1A and the root development regulator MeLHW, MeCEPD coordinates carbon–nitrogen balance and root architecture remodeling. These findings reveal a novel TGA-independent regulatory module in cassava and provide potential targets for improving nitrogen use efficiency in crops.

Future studies should focus on elucidating the upstream signals activating *MeCEPD* under nitrate deficiency and the mechanistic basis by which its interactions with MeRBCS1A and MeLHW integrate nitrogen signaling with carbon metabolism and root development.

## Data Availability

The original contributions presented in this study are included in the article/Supplementary Materials. Further inquiries can be directed to the corresponding authors.

## Supplementary Materials

The following supporting information can be downloaded at https://www.mdpi.com/article/10.3390/plants15071056/s1. Supplementary Table S1. Features of *Arabidopsis* CC-type GRX genes; Supplementary Table S2. Genomic characteristics of cassava CC-type GRX genes; Supplementary Table S3. Mutation analysis of *MeCEPD* CRISPR/Cas9-edited cassava lines; Supplementary Table S4. Primers used in this study; Supplementary Figure S1. Analysis of cis-acting elements in promoters of cassava CC-type GRX genes; Supplementary Figure S2. CRISPR/Cas9 targeting strategy for *MeCEPD* and expression analysis of overexpression lines.

## Abbreviations

The following abbreviations are used in this manuscript:

GRX	Glutaredoxin
CEP	C-terminally encoded peptide
CEPD	Polypeptides CEP DOWNSTREAM
TGA	TGA G box-binding factor
RBCS1A	Rubisco small subunit 1A
LHW	LONESOME HIGH WAY
Ura	Uracil
Leu	Leucine
RD21B	Responsive to dehydration 21B
Co-IP	Co-immunoprecipitation
LCI	Luciferase complementation imaging
MEME	Multiple EM for Motif Elicitation
X-α-gal	5-bromo-4-chloro-3-indolyl-α-D-galactopyranoside
MS	Murashige and Skoog medium
FPKM	Fragments per kilobase of transcript per million mapped reads

## Appendix A

**Table A1:** Cis-element analysis of *MeCEPD* promoter.

Name	Type	Sequence	Number
Light-responsive elements	G-BOX	CACGTG	4
	AAAC	CAATCAAAACCT	1
	GATA	CTCCTGATTAGC	1
	AE-box	AGAAACTT	1
	TCCC	TCTCCCT	1
Abiotic stress-responsive element	MBS	(T/C)C(T/C)AACGG(T/C)(T/C)A	1
	LTR	CCGAAA	1
	MYB	CAACCA	2
	Myb	CAACTG	1
Hormone-responsive elements	ABRE	GACACGTACGT	1
		ACGTG	2
		CACGTG	2
		CGTACGTGCA	2
Cell cycle regulation-responsive elements	MSA-like	(T/C)C(T/C)AACGG(T/C)(T/C)A	1
Seed-specific regulation-responsive elements	RY	CATGCATG	1
