# Supplementary material for: CC-Type Glutaredoxin MeCEPD Functions as an Important Regulatory Component in Response to Nitrate Starvation in Cassava
Source: Plants (Basel). 2026 Mar 30;15(7):1056. doi: 10.3390/plants15071056 (PMC13074788; doi:10.3390/plants15071056)
Supplement: Supplementary file 1 [file plants-15-01056-s001.zip › Supplementary Figure.pdf]

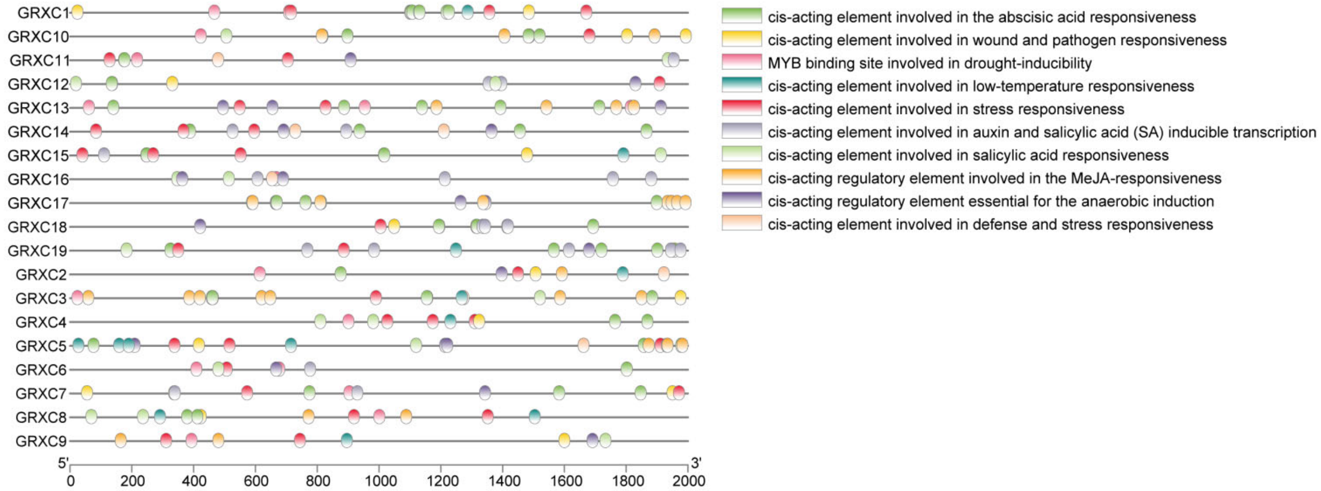

Supplementary Figure S1. Schematic diagram showing the positions of stress- and hormone-related cis-elements in the 2000 bp promoter regions of 19 cassava GRX genes. Elements were predicted using the PlantCARE database. Elements related to phytohormone signaling (abscisic acid, MeJA, and salicylic acid responsiveness) and stress responses (wound/pathogen, defense, drought, low-temperature, and anaerobic induction) are indicated by colored boxes at their respective positions. The distribution patterns reveal that *MeCEPD* contains multiple ABA-responsive elements but lacks MeJA- and SA-responsive elements present in other family members.

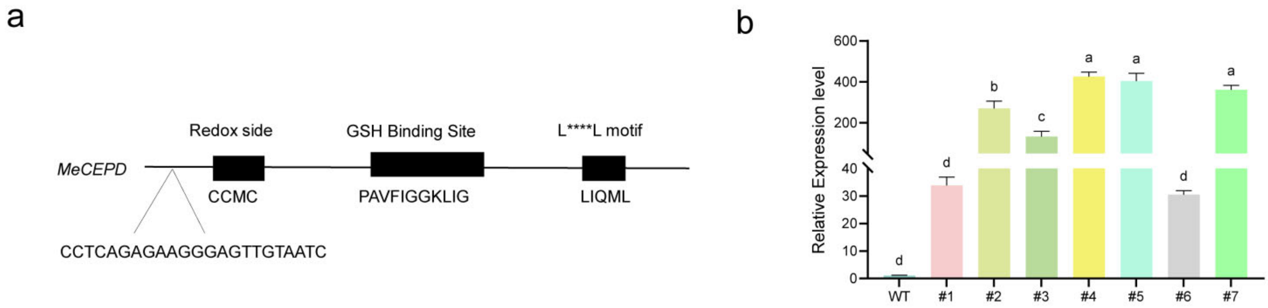

Supplementary Figure S2. CRISPR/Cas9 targeting strategy for *MeCEPD* and expression analysis of overexpression. (a) Conserved domains (including the CCMC active site) are indicated by black boxes. The target sequence (23 bp) is located upstream of the CCMC active site. (b) RT-qPCR analysis of *MeCEPD* expression in seven independent overexpression lines. Expression levels are shown as fold change relative to WT (mean ± s.d., n = 3). Data were analyzed by one-way ANOVA followed by Tukey's post-hoc test; different letters indicate significant differences ($P < 0.05$). Overexpression line #2 (designated CEPDox) was selected for phenotypic analysis.
