# Supplementary material for: CC-Type Glutaredoxin MeCEPD Functions as an Important Regulatory Component in Response to Nitrate Starvation in Cassava
Source: Plants (Basel). 2026 Mar 30;15(7):1056. doi: 10.3390/plants15071056 (PMC13074788; doi:10.3390/plants15071056)
Supplement: Supplementary file 1 [file plants-15-01056-s001.zip › Supplementary Table.pdf]

**Supplementary Table S1.** Features of *Arabidopsis* CC-type GRX genes

| Gene Name | TAIR ID | Chromosomal Location | Introns | Protein (aa) | ALWL motif | Redox Site |
|---|---|---|---|---|---|---|
| AtROXY1 | AT3G02000 | Chr3:332275 - 333072 reverse | 0 | 136 | ALWL | CCMC |
| AtROXY2 | AT5G14070 | Chr5:4541780 - 4542585 forward | 0 | 140 | ALWL | CCMC |
| AtROXY3 | AT3G21460 | Chr3:7557297 - 7557969 reverse | 0 | 102 | AIWL | CCMS |
| AtROXY4 | AT3G62950 | Chr3:23266227 - 23267090 forward | 0 | 103 | AIWL | CCMS |
| AtROXY5 | AT2G47870 | Chr2:19603163 - 19603895 forward | 0 | 103 | AIWL | CCMC |
| AtROXY6 | AT1G06830 | Chr1:2097106 - 2097655 forward | 0 | 99 | - | CCLC |
| AtROXY7 | AT2G30540 | Chr2:13011105 - 13012043 reverse | 0 | 102 | - | CCMS |
| AtROXY8 | AT3G62960 | Chr3:23268641 - 23269324 forward | 0 | 102 | - | CCLC |
| AtROXY9 | AT2G47880 | Chr2:19605030 - 19605704 forward | 0 | 102 | - | CCLC |
| AtROXY10 | AT5G18600 | Chr5:6183166 - 6184038 reverse | 0 | 102 | ALWV | CCMS |
| AtROXY11 | AT4G15700 | Chr4:8937358 - 8938124 forward | 0 | 102 | ALWL | CCMS |
| AtROXY12 | AT4G15690 | Chr4:8934296 - 8934961 forward | 0 | 102 | ALWL | CCMS |
| AtROXY13 | AT4G15680 | Chr4:8931617 - 8932317 forward | 0 | 102 | ALWL | CCMS |
| AtROXY14 | AT4G15670 | Chr4:8929031 - 8929813 forward | 0 | 102 | ALWL | CCMS |
| AtROXY15 | AT4G15660 | Chr4:8925571 - 8926510 forward | 0 | 102 | ALWL | CCMS |
| AtROXY16 | AT1G03020 | Chr1:697919 - 698591 reverse | 0 | 102 | AIWI | CCMS |
| AtROXY17 | AT3G62930 | Chr3:23261442 - 23261927 reverse | 0 | 102 | AIWV | CCMS |
| AtROXY18 | AT1G03850 | Chr1:975880 - 977761 reverse | 1 | 159 | ALWL | CCLG |
| AtROXY19 | AT1G28480 | Chr1:10013434 - 10014256 reverse | 0 | 137 | ALWL | CCMC |
| AtROXY20 | AT5G11930 | Chr5:3844940 - 3845711 reverse | 0 | 148 | ALWA | CCMC |
| AtROXY21 | AT4G33040 | Chr4:15940228 - 15941493 reverse | 0 | 144 | ALWV | CCMC |

Note: Gene names, TAIR IDs, chromosomal locations, intron numbers, protein lengths, ALWL motifs, and redox sites were obtained from The Arabidopsis Information Resource (TAIR, https://www.arabidopsis.org/). All information was accessed on 5 March 2026.

**Supplementary Table S2.** Genomic characteristics of cassava CC-type GRX genes

| Gene Name | Gene ID | Chromosomal Location | Gene Size (bp) | CDS Size (bp) | Introns | Protein (aa) | GenBank Accession |
|---|---|---|---|---|---|---|---|
| MeGRXC1 | 110610704 | Chromosome01:38437461..38437767 reverse | 306 | 306 | 0 | 101 | OAY61766.1 |
| MeGRXC2 | 122725064 | Chromosome01:38438704..38440172 reverse | 1468 | 309 | 0 | 102 | OAY61767.1 |
| MeGRXC3 | 110626586 | Chromosome01:38466742..38467618 forward | 876 | 315 | 0 | 104 | OAY61770.1 |
| MeGRXC4 | 110612295 | Chromosome01:38471128..38472715 forward | 1587 | 309 | 1 | 102 | OAY61771.1 |
| MeGRXC5 | 110611124 | Chromosome03:4541545..4542964 reverse | 1419 | 405 | 0 | 134 | OAY54116.1 |
| MeGRXC6 | 110612267 | Chromosome03:31062196..31063084 forward | 888 | 306 | 0 | 101 | OAY55956.1 |
| MeGRXC7 | 110615985 | Chromosome05:5557615..5558332 reverse | 717 | 318 | 0 | 105 | OAY49575.1 |
| MeGRXC8 | 122723640 | Chromosome05:5570681..5570990 forward | 309 | 309 | 0 | 102 | OAY49579.1 |
| MeGRXC9 | 122723641 | Chromosome05:5571289..5573663 forward | 2374 | 306 | 2 | 101 | OAY49581.1 |
| MeGRXC10 | 110627019 | Chromosome11:12471971..12472930 forward | 959 | 372 | 0 | 123 | OAY37215.1 |
| MeGRXC11 | 110628837 | Chromosome12:787832..788970 forward | 1138 | 417 | 0 | 138 | OAY34256.1 |
| MeGRXC12 | 110629644 | Chromosome13:1156919..1157336 forward | 417 | 306 | 0 | 101 | OAY32295.1 |
| MeGRXC13 | 110629449 | Chromosome13:35496613..35497084 forward | 471 | 471 | 0 | 156 | OAY33994.1 |
| MeGRXC14 | 110602362 | Chromosome15:1271064..1271370 reverse | 306 | 306 | 0 | 101 | OAY27782.1 |
| MeGRXC15 | 110602361 | Chromosome15:1274727..1275620 forward | 893 | 432 | 1 | 143 | KAG8636592.1 |
| MeGRXC16 | 110600791 | Chromosome15:9878625..9879744 forward | 1119 | 417 | 0 | 138 | KAG8637451.1 |
| MeGRXC17 | 110603366 | Chromosome16:3992574..3993373 reverse | 799 | 447 | 0 | 148 | OAY26248.1 |
| MeGRXC18 | 110603335 | Chromosome16:28782327..28782762 reverse | 435 | 435 | 0 | 144 | OAY26869.1 |
| MeGRXC19 | 110605518 | Chromosome17:24747525..24748370 reverse | 845 | 453 | 0 | 150 | OAY24868.1 |

Note: Gene IDs and GenBank accession numbers were obtained from the NCBI database (https://www.ncbi.nlm.nih.gov/gene/). Chromosomal locations, gene sizes, CDS sizes, intron numbers, and protein lengths were retrieved from the Phytozome database (v14, Manihot esculenta v8.1, https://phytozome-next.jgi.doe.gov/). All information was accessed on 4 March 2026.

**Supplementary Table S3.** Mutation analysis of *MeCEPD* CRISPR/Cas9-edited cassava lines.

| Lines | Sequence 5′-3′ | Mutation type |
|---|---|---|
| WT | CCTCAGAGAAGGGAGTTGTAATC | |
| #2 | CCTCAGa**AGGGAGTTGTAATC | -2 |
| #4 | CCTCAGa**AGGGAGTTGTAATC<br>CC**********************TC | -2/-22 |
| #5 | CCTCAGa**AGGGAGTTGTAATC<br>CCTCAGAAGAAGGGAGTTGTAATC<br>CCTCAGTAGAAGGGAGTTGTAATC | -2/+1/+1 |
| #23 | CCTCAGAAGAAGGGAGTTGTAATC<br>CCTCAGTAGAAGGGAGTTGTAATC | +1/+1 |
| #25 | CCTCAGa**AGGGAGTTGTAATC<br>CCTCAgTAGAAGGGAGTTGTAATC | -2/+2 |

Note: WT, wild-type; #, edited lines. Red text indicates the PAM-binding site; yellow text indicates edited sequences. Asterisks (*) represent nucleotide deletions. Lowercase letters indicate inserted or mutated nucleotides. Plus sign (+) in mutation type indicates nucleotide insertion. For each line, multiple rows represent different alleles. Line #2 carries a homozygous 2-bp deletion. Lines #4, #23, and #25 are biallelic mutants carrying different mutations in each allele. Line #5 is a chimera with three alleles. Line #2 was selected as the representative *cepd* line for phenotypic analysis in this study.

**Supplementary Table S4.** Primers used in this study

| Primer Name | Sequence 5′-3′ | Purpose |
|---|---|---|
| MeCEPD realtime-F | GACCCTGAAGGCAGAGAAATG | RT-qPCR |
| MeCEPD realtime-R | GGTGCCACTTAGATGGTGTG | RT-qPCR |
| MeGRXC2 realtime-F | GCTGCAGTTATCTTCACCAAG | RT-qPCR |
| MeGRXC2 realtime-R | TGCCTTAGCATCTATGAGCATT | RT-qPCR |
| MeGRXC4 realtime-F | CGAGTTGGTTAAGGAAAAGC | RT-qPCR |
| MeGRXC4 realtime-R | CTCGTAAACTGTCGGATTGG | RT-qPCR |
| MeGRXC8 realtime-F | GGATAGGGTGAGAGATTTGGC | RT-qPCR |
| MeGRXC8 realtime-R | CCTATGAACACAGCTGGGAC | RT-qPCR |
| MeGRXC9 realtime-F | GAGATTGACCAAGACACTGAAG | RT-qPCR |
| MeGRXC9 realtime-R | CCTCCATCTTAGTGGCACAC | RT-qPCR |
| MeGRXC15 realtime-F | AGAGTTACGCAATTGGCATC | RT-qPCR |
| MeGRXC15 realtime-R | GCTTGTTCTATTTCCCTTCC | RT-qPCR |
| MeTGA1 realtime-F | ATCCACCAGATTGGCATGTG | RT-qPCR |
| MeTGA1 realtime-R | GACGTCTTTGTACCTGTAAACACT | RT-qPCR |
| MeNRT1.1 realtime-F | TCAAATCAAGTGTCTCGGGC | RT-qPCR |
| MeNRT1.1 realtime-R | GTGCCAGACAAGAACAGGAT | RT-qPCR |
| MeNRT2.1 realtime-F | TATTTCCGGCATGACTGGTG | RT-qPCR |
| MeNRT2.1 realtime-R | TCTTCGGTGGATTTCACGAC | RT-qPCR |
| MeNRT2.4 realtime-F | GTTCTGGACTGACACAGCTT | RT-qPCR |
| MeNRT2.4 realtime-R | TCTGCTTTTCCTCCTCGTTC | RT-qPCR |
| MeNRT3.1 realtime-F | TCCGGAAAACTCTTGTGGTC | RT-qPCR |
| MeNRT3.1 realtime-R | TTGGATAGGTTGTCCTCGGT | RT-qPCR |
| MeNIA realtime-F | AAGGTCCACTGGGTCACATA | RT-qPCR |
| MeNIA realtime-R | CAAGCATCAAGCTCGTCTCT | RT-qPCR |
| MeNIR realtime-F | GCTAGAGCCATGAAGGTGAC | RT-qPCR |
| MeNIR realtime-R | CCTCCCAAGAACACATCCAC | RT-qPCR |
| MeRBCS1A realtime-F | AGGGTACTACGATGGACGTT | RT-qPCR |
| MeRBCS1A realtime-R | AACTGATGCACTGGACTTGG | RT-qPCR |
| MeLHW realtime-F | GTTGAACTTCTTGTGGCTGAC | RT-qPCR |
| MeLHW realtime-R | TTCTTCTGGACACTTGGGAATTA | RT-qPCR |
| MeRBCS1A ADm-F | GCGTCGACATGGCTACCTCTATGCTTTCAACTG | Y2H |
| MeRBCS1A ADm-R | CGCGGATCCATAATCGGTGCCGGGAGG | Y2H |
| MeLHW ADm-F | GCGTCGACATGGGAACTACTGCTTTGAGGC | Y2H |
| MeLHW ADm-R | CGCGGATCCAATCTTGCTCGAGATAGGACTCC | Y2H |
| MeRBCS1A ADm-F | GCGTCGACATGGCTACCTCTATGCTTTCAACTG | Y2H |
| MeRBCS1A ADm-R | CGCGGATCCATAATCGGTGCCGGGAGG | Y2H |
| MeCEPD BDm-R | ACGCGTCGACATGGAAAAGGTGATGGGACTGG | Y2H |
| MeCEPD BDm-R | ACGCGTCGACAGATAAAGTCTGGTATGGCTTGAGC | Y2H |
| MeCEPD Cas9-F | GATTGATTACAACTCCCTTCTCTG | CRISPR |
| MeCEPD Cas9-R | AAACCAGAGAAGGGAGTTGTAATC | CRISPR |
